# Forecasting study of food-related patents protected by the University of Brasilia, Brazil: Case study

**DOI:** 10.1016/j.heliyon.2023.e17111

**Published:** 2023-06-08

**Authors:** Rafael França Neves, Marileusa Dosolina Chiarello, Larisse Araújo Lima, Grace Ferreira Ghesti

**Affiliations:** a Nutrition Department, University of Brasilia, Campus Darcy Ribeiro, 70910-900, Brasilia, Brazil; b Technology Development Center, University of Brasilia, Campus Darcy Ribeiro, 70910-900, Brasilia, Brazil; c Laboratory of Brewing Bioprocesses Technology and Catalysis in Renewable Energy, Institute of Chemistry, University of Brasilia, Campus Darcy Ribeiro, 70910-900, Brasilia, Brazil

**Keywords:** University of Brasilia, Patents, Food, Innovation

## Abstract

Patents are important tools to protect innovations in the food industry. They are also indicators of the research and development of an institution. Universities play a significant role in generating and developing innovative technologies. Center for Support to Technological Development (CDT is the Technological innovation center (NIT) that is responsible for protecting the technologies developed by the academic community of the University of Brasilia (UnB). This case study analyzes the patents and patent applications related to food deposited by the UnB. For this purpose, a search was conducted on the National Institute of Industrial Property (INPI) institutional page through the patent database. The results show diverse applications to the food area that are mainly related to biotechnology. Half of the protections are still in progress in the INPI workflow. The requirements issued were primarily related to national genetic heritage. Archiving processes were identified due to non-payment, but we highlighted that it could be a strategic decision of NIT/CDT. Rejections were mainly related to the lack of novelty or inventive steps. Currently, two food-related patents are in force that took, on average, nine and a half years to be granted. Although UnB exclusively owns the majority, the results also evidence co-ownership with other universities and companies. Finally, this study highlights possible partnerships between UnB and the food industry through technology transfer. The technology transfer indicators pointed out that UnB has expertise in this area and that there is a potential to be explored. These results contribute to strategic decision-making in developing new technologies related to food and nutrition and their transfer to society. The scale-up and the increased degree of maturity require greater interaction with the productive sector to ensure the transfer of technology for innovation arising from the research conducted at UnB.

## 1 Introduction

Intellectual property refers to the protection of human mind creations. It applies to various activities and is essential to humankind’s technological, economic, cultural, and social progress. The importance of intellectual property is recognized by several national and international laws and agreements that protect different intellectual property rights. These laws provide creators and inventors with the legal expression of intellectual property rights, balancing the public interest in accessing protected knowledge [1].

Industrial property is the branch of intellectual property focused on business activity, including industry, commerce, and service provision [2]. In Brazil, the National Institute of Industrial Property (INPI) is the federal agency, linked to the Ministry of Economy (ME), responsible for granting industrial property rights that contemplates the protections related to patents, industrial designs, trademarks, and recognition of geographical indications. These protections are governed by Law No. 9279 of May 14, 1996, which regulates the rights and obligations relating to industrial property [3].

According to the Brazilian Food Industry Association (ABIA), the Brazilian food and beverage industry is the largest, representing 10.6% of the Brazilian Gross Domestic Product (GDP) and generating 1.68 million formal and direct jobs. Worldwide, Brazil is the second-largest exporter of processed foods [4]. The protection of intangible assets of high added value, such as trademarks, industrial designs, geographical indications, and patents, has a strong presence in the food industry and are factors related to the expressive dimension of this industry in the national and international scenario [5]. However, the intellectual property system may not be very effective for the food industry. Consequently, the food industry works extensively with industrial secrets (know-how) to differentiate itself and avoid competition, often for not meeting the protection requirements [6].

In the scope of industrial property in the food industry, patents are important tools to foster innovations since they make it possible to protect the innovative technical character of a specific product, process, or use [5]. This type of protection can be understood as a temporary property title granted to inventors or other holders of rights over creating a particular invention valid in a specific territory. With the title, the inventor or patent holder has the right to prevent others from producing, using, marketing, or importing the product or process of his patent without his consent. On the other hand, the inventor must disclose in detail the entire technical content of the protected subject matter [3].

Although the scientific literature has long considered the food industry as a sector with low innovation intensity, in recent years, the application of new technologies to meet the constant transformations of the external environment has promoted a considerable increase in innovation in the sector, making this an essential tool to ensure market competitiveness [7]. In this context, patent protection for innovative products and processes generated within the food industry and related industries can represent an important market strategy [5].

Meanwhile, Universities represent one of the main poles of knowledge generation and development of innovative technologies eligible for patent protection, including food-related ones. This process developed in universities allows it to be transmitted more effectively to society and provides a greater appreciation of researchers and increased motivation for academic careers [8].

Technological innovation centers (NITs) are organizations created to manage innovation policy within universities [9]. At the University of Brasília (UnB), the Center for Support to Technological Development (CDT) is responsible for assuming the NIT functions foreseen in the legislation [10]. These functions include promoting and monitoring the relationship of Science and Technology Institutions (ICTs) with companies, managing intellectual property protection and technology transfer processes, and supporting entrepreneurship and innovation [9,[11], [12], [13]].

Within NIT/CDT, the Coordination of Innovation and Technology Transfer (CITT) through the Intellectual Property Nucleus (Nupitec) is the area responsible for protecting the technologies developed by the academic community of the UnB [14]. Regarding these technologies, it can be highlighted that patents and patent applications are intellectual property with the highest number of protections [15].

Thus, the general objective of this work is to analyze intangible assets protected in the form of patents owned by UnB related to food. The specific objectives are to identify trends and opportunities related to food innovation at the UnB, to discuss the role of the NIT/CDT in the management of intangible assets and enforcement of industrial property rights, and to elucidate which are the possible partnerships between the University and the food industry in the development of new technologies through the NIT/CDT.

This case study aims to contribute to strategic decision-making in the development of new technologies related to the area of food and nutrition and their transfer to society, increasing the degree of confidence from an approach based on data and evidence to promoting better availability of financial, human, administrative, marketing and material resources for research with the potential to generate protectable assets within the University. The study is justified since patents are important indicators of an institution’s research and development. It is possible to measure aspects of inventive activity and explore the knowledge and application of industrial property rights by analyzing patent documents.

## 2 Methodology

The case study is a methodology that aims to understand complex social phenomena, such as innovations and intellectual production in universities. This methodology seeks to understand and, at the same time, develop theories concerning the observed phenomenon. Thus, the case study is characterized as a method suitable for answering the “how” and “why” questions [16]. Therefore, the guiding question was, “How are food-related patent protections established at the University of Brasilia (UnB)?". The case study in question has a descriptive purpose from a quantitative approach of patents and patent applications related to food deposited by the University.

All assets protected in the form of patents requested by the University of Brasilia Foundation (*Fundação Universidade de Brasília* - FUB), legal entity that represents the UnB patent requests, related to products and processes involving food, ingredients, food additives, food quality control, machines and tools for food processing, microorganisms and enzymes for food production, polymers, and food packaging were included. Patent assets on quality control, processing machines and tools, microorganisms and enzymes, polymers and packaging when not related to food, and assets related to pharmaceuticals, pest control, animal fodder, tobacco, metallurgy, textiles, paper, fixed constructions, mechanical engineering, lighting, heating, weapons, explosion, physics, electricity, and other non-food related were excluded.

Once the direction of the research was defined, a search was conducted on the institutional page of the INPI through the patent database. The searches in this database allow access to all patent applications filed in Brazilian territory, provided they are not under confidentiality. The database can be accessed through INPI’s website <www.inpi.gov.br>. The “Advanced Search” option has five search fields, each with specifications for building a search strategy.

For the search in question, on the tab “Depositor/owner/inventor” in the field Name of the Depositor/owner, “*Fundação Universidade de Brasília*", was entered. The depositor’s CPF/CNPJ, “00038174000143″, was entered to retrieve only patents and patent applications filed by FUB. The search was done by combining the items' filling out. The fields “Application No.”, “Country/Priority No.”, “Filing No. (PCT), Filing Date”, “Priority Date, Filing Date (PCT)", “Publication Date (PCT)", “IPC Classification”, “Keyword in IPC classifier”, “Title”, “Abstract”, and “Inventor Name” was not filled in. This search was conducted on 12/10/2021.

After conducting the complete search in the INPI database, all identified records were collected and organized in a spreadsheet prepared in the Microsoft® Excel® program. The patent applications in the confidentiality period were removed before the screening, as they did not bring necessary information for screening, such as title, abstract, and International Patent Classification (IPC). Each record was assessed by title, then abstract concerning its eligibility. In each step, records that did not meet the inclusion criteria were excluded from this study. The search results were presented in an adaptation of the Preferred Reporting Items for Systematic Reviews and Meta-analyses (PRISMA) flow chart [17].

The patents that met the inclusion criteria were evaluated and detailed in a tabular form aligned with the study’s objective. The included patents were analyzed regarding the request and its application in the food area, the technological field, the claims, the processing, and the applicants/owners. The documents analyzed to extract this information included information available on the patent application homepage, in the publication of the applications, in the rejection opinions, and in the granted patents and other associated documents, when relevant.

## 3 Results and discussion

**Fig. 1:**
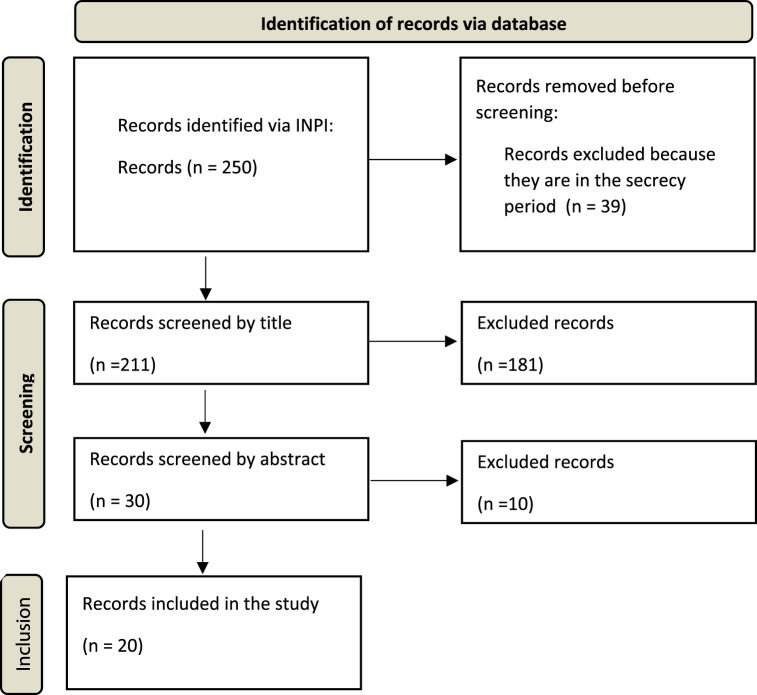
Quantitative representation of the record selection process.

The initial search found two hundred fifty records in the INPI patent database. All retrieved records are from a national invention patent application filing. After excluding the records in the confidentiality period, 211 records remained. From the screening by reading the titles, 181 records were excluded; after reading the abstracts, another ten records were excluded. After the screening, 20 records were included. Notably, no application for a utility model patent or certificate of addition related to food was found. The quantitative representation of the record selection process can be found in Fig. 1.

**Fig. 2:**
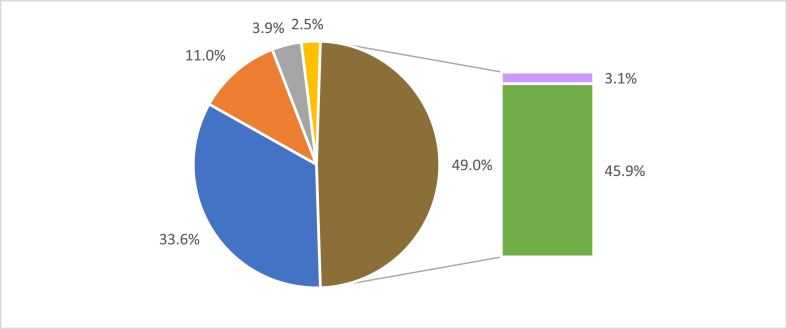
Participation of food-related patents and patent applications. Computer programs (33.6% - color blue); Trademarks (11.0% - color orange); Industrial designs (3.9% - color grey); Cultivars (2.5% - color yellow); National patents and patent applications related to food (3.1% - color pink); Other national and international patents/patent applications (45.9% - color green). (For interpretation of the references to color in this figure legend, the reader is referred to the Web version of this article.)

Patents and patent applications are the predominant protection modality in ICTs [18]. For example, UnB presents a predominance of patent protection, followed by computer programs, trademarks, and industrial designs [19]. Considering the total of 316 patents/patent applications protected by the NIT/CDT (250 national and 66 international), those related to foodstuffs correspond to only 8.00% of the federal protections and 6.32% of the total protections in the patent modality. The participation of food-related patents and patent applications in the total of protections at the UnB is only 3.1%, as shown in Fig. 2.

It is possible to observe that the participation of food-related patents is not yet very expressive within the UnB. Courses such as Agronomy, Biological Sciences, Pharmaceutical Sciences, and Nutrition have been responsible for producing food-related patents at the University. However, UnB does not have Food Engineering and Food Science courses, which in other universities play a significant role in this field of patent. These data demonstrate the necessity of disseminating the culture of intellectual property among researchers and students in the area to direct resources to research and develop new products and processes that patents can protect. The protection of knowledge developed in universities allows it to be transmitted more effectively to society, provides a greater appreciation for researchers, and increases motivation for academic careers [8].

Critical data were extracted from the included records, such as application number, the full title of the application, IPC classification, claims, processing, and holders. These data were organized in tabular form in Table 1.

**Table 1:** Characterization of the retrieved records.

Request No.	Title	IPC∗	Claims	Workflow	Depositor
BR 10 2019 024756 8	Recombinant yeast for pullulanase production, its obtaining process, and use	C12 N 1/19	Product, process, and use	Deposit: 11/25/19 Receipt: 03/12/19 Publication: 08/06/21	*Federal University of São João Del Rei* (BR/MG)/*University of Brasilia Foundation* (BR/DF)
BR 10 2019 023411 3	A genetically modified strain of *Saccharomyces cerevisiae* for the production of long-chain polyunsaturated fatty acids, its production process, and its use applied to industry	C12 N 1/19	Product, process, and use	Deposit: 11/07/19 Receipt: 11/19/19 Demand: 02/11/20 Notification: 03/03/20 Publication: 05/18/21	*University of Brasilia Foundation* (BR/DF)
BR 10 2019 023405 9	A genetically modified strain of *Saccharomyces cerevisiae* for the production of short and medium-chain fatty acids, its obtaining process, and its use applied to industry	C12 N 1/18	Product, process, and use	Deposit: 11/07/19 Receipt: 11/19/19 Demand: 02/11/20 Notification: 03/03/20 Publication: 05/18/21	*University of Brasilia Foundation (*BR/DF)
BR 10 2019 020528 8	2′-fucosyllactose production by a recombinant strain of *Kluyveromyces lactis* using mannose as substrate	C12 N 1/19	Product, process, and use	Deposit: 09/30/19 Receipt: 08/10/19 Requirement: 12/31/19 Notification: 04/02/20 Publication: 04/20/21	*University of Brasilia Foundation* (BR/DF)/*National Institute of Technology - INT* (BR/RJ)
BR 10 2018 067854 0	Alginate-chitosan microspheres with fungal amylases immobilized in an external phase, their obtainment process, and their use in starch hydrolysis into reducing sugars	C12 N 11/00	Product, process, and use	Deposit: 09/05/18 Receipt: 09/25/18 Notification: 12/11/18 Publication: 03/17/20	*University of Brasilia Foundation* (BR/DF)
BR 10 2018 067855 8	Alginate-chitosan microspheres with fungal amylases immobilized in a single phase, their production process, and their use in the hydrolysis of starch into reducing sugars	C12 N 11/00	Product, process, and use	Deposit: 09/05/18 Receipt: 09/25/18 Notification: 12/11/18 Publication: 03/17/20	*University of Brasilia Foundation* (BR/DF)
BR 10 2018 067853 1	Alginate-chitosan microspheres with fungal amylases immobilized in an internal phase, their obtainment process, and their use in the hydrolysis of starch into reducing sugars	C12 N 11/00	Product, process, and use	Deposit: 09/05/18 Receipt: 09/25/18 Notification: 12/11/18 Publication: 03/17/20	*University of Brasilia Foundation* (BR/DF)
BR 10 2018 067395 5	Virgin avocado oil extraction process	C11B 1/04	Process and use	Deposit: 08/31/18 Receipt: 09/25/18 Notification: 11/27/18 Publication: 03/10/20	*University of Brasilia Foundation* (BR/DF)
BR 10 2018 011822 6	Single-chamber reactor and process for fluidized-bed grain malting	C12C 1/00	Product and process	Deposit: 06/11/18 Receipt: 06/19/18 Notification: 10/02/18 Publication: 12/24/19 Archived: 09/21	*University of Brasilia Foundation* (BR/DF)
BR 10 2018 011677 0	Processes for obtaining modified cellulose - carboxylase - and its applications as an additive	C08L 1/02	Product, process, and use	Deposit: 06/08/18 Receipt: 06/19/18 Demand: 09/25/18 Notification: 10/23/18 Publication: 12/24/19 Archived: 09/21	*University of Brasilia Foundation* (BR/DF)
BR 10 2017 025294 9	Nanostructured composition comprising vegetable oil derived from species of the genus *Caryocar* and its applications	A61K 36/185	Product, process, and use	Deposit: 11/24/17 Receipt: 12/05/17 Notification: 06/26/18 Publication: 06/11/19	*University of Brasilia Foundation* (BR/DF)
BR 10 2016 020328 7	Antioxidant microparticles and their uses	A23P 10/30	Product and use	Deposit: 08/31/16 Receipt: 06/27/17 Notification: 08/29/17 Publication: 03/20/18 Formal Requirement: 12/26/18 Preliminary Demand: 12/17/19	*State University of Campinas* - Unicamp (BR/SP)/*University of Brasilia Foundation* (BR/DF)
BR 10 2014 031451 2	Process for obtaining a quinoa base product for food, quinoa base product for food, quinoa beverage	A23L 25/00	Product, process, and use	Deposit: 12/16/14 Receipt: 12/29/15 Notification: 03/05/16 Publication: 09/27/16 Formal Requirement: 02/27/18 Preliminary Demand: 08/09/20 Grant: 06/04/21 Concession: 07/13/21	*University of Brasilia Foundation* (BR/DF)
BR 10 2012 021044 4	Selol-based composition in micro and nanoemulsions, its production process, its applications in the prevention and treatment of neoplasms, and food supplementation	A61K 31/095	Product, process, and use	Deposit: 08/22/12 Check-in: 01/29/13 Notification: 12/23/14 Publication: 03/10/15 Archive: 01/24/17 Restoration: 05/23/17 Notice: 06/03/18 Formal Requirement: 03/27/18 Notice of Consent: 12/17/19 Preliminary Demand: 06/30/20 Knowledge of Technical Opinion: 12/29/20 Rejection: 04/13/21 Maintenance of Rejection: 06/29/21	*University of Brasilia Foundation* (BR/DF)
PI 0903673-3	Green banana flour-based gluten-free pasta, production process, and application	A21D 2/36	Product, process, and use	Deposit: 11/09/09 Notification: 02/23/10 Publication: 10/05/11 Archiving: 02/14/17 Restoration: 05/23/17 Change of Classification: 03/27/18 Formal Requirement: 04/17/18 Awareness of Technical Opinion: 12/18/18 Rejection: 03/26/19 Maintenance of Rejection: 06/11/19	*University of Brasilia Foundation* (BR/DF)
PI 0902864-1	Proteins, protease inhibitor peptides and their derivatives, drug compositions, nutraceuticals, and cosmeceuticals based on these compounds and their uses in the preparation of formulations for the treatment and prevention of cancer or diseases involving protease inhibition	C07K 14/81	Product, process, and use	Deposit: 07/17/09 Notification: 12/01/09 Publication: 03/15/11 Filing: 01/22/13 Archive: 06/25/13 Classification Amendment: 03/27/18	*University of Brasilia Foundation* (BR/DF)
PI 0805516-5	Extracts and their derivatives from plants of the genus *Pouteria*, processes for obtaining them, and their use in compositions with therapeutic, cosmetic, or nutraceutical action	A61K 36/185	Product, process, and use	Deposit: 12/02/08 Notification: 05/19/19 Publication: 08/24/10 Communication: 09/27/16 Archive: 02/14/17 Restoration:05/23/17 Notification:08/08/17 Awareness of Technical Opinion: 10/17/17 Rejection: 04/03/18 Maintenance of Rejection: 06/19/18	*University of Brasilia Foundation* (BR/DF)
PI 0803149-5	Recombinant bovine pro-chymosin gene and its expression in fungi aiming at the production of cheese and its derivatives	C12 N 15/59	Product, process, and use	Deposit: 07/31/08 Notification: 06/01/09 Publication: 06/08/10 Demand: 04/24/12 Filing: 03/01/17 Restoration: 05/23/17 Awareness of Technical Opinion: 10/10/17 Rejection: 04/03/18 Maintenance of Rejection: 06/19/18	*University of Brasilia Foundation* (BR/DF)/*Coalhos Bio Paraná Ltda* (BR/PR)
PI 0601631-6	Jelly capsules of pequi (*Caryocar brasiliense camb*) pulp as vitamin, antioxidant and antimutagenic supplement, a new nutraceutical	A61K 9/48	Product, process, and use	Deposit: 01/31/06 Notification:20/06/06 Publication:06/11/07 Archive: 12/20/16 Restoration: 05/23/17 Notice: 06/03/18 Formal Requirement: 04/24/18 Notification: 07/02/19 Preliminary Demand: 03/31/20 Archived: 06/16/20	*University of Brasilia Foundation* (BR/DF)
PI 0000383-2	Hybrid milk pasteurizer - slow pasteurization.	A23L 3/00	Product	Deposit: 02/03/00 Entered: 11/28/00 Publication: 09/25/01 Grant: 03/20/12 Grant: 09/18/12 Call for restoration: 06/25/13 Order canceled: 07/16/13	*University of Brasilia Foundation* (BR/DF)

∗ Subtitle for IPC classification: see supplementary material.

### 3.1 Application to the food area

The profile of the retrieved applications is quite heterogeneous due to the specific nature of each invention. In the general scope, the protected objects refer to products and processes related to genetically modified microorganisms, extraction, and processing of plant species or chemical synthesis with various applications in the food industry. Some of these industrial applications include those related to the processing of starch, in particular for the production of glucose and other sugars; to the production of milk, cheese, and other dairy products; to applications as food additives and processing aids, food ingredients with functional and health claims, food supplements, special purpose foods, and other nutraceutical claims.

### 3.2 Technological field

The IPC classification provides a hierarchical system for classifying patents according to the different areas of technology to which they belong. Thus, it can be used as a primary categorization tool for the technological areas of food patenting [20]. A classification developed by the World Intellectual Property Organization (WIPO) allows relating the IPC codes to the corresponding technological fields, enabling the identification of the fields and sectors related to the intellectual production of patent applications [21,22].

**Fig. 3:**
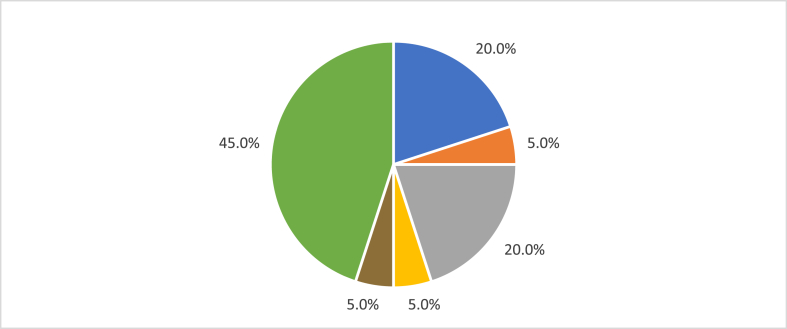
Distribution of assets by technological field. Pharmaceuticals (20.0% - color blue); Macromolecular chemistry, polymers (5.0% - color orange); Food chemistry (20.0% - color grey); Chemistry of basic materials (5.0% - color yellow); Other special machines (5.0% - color brown); Biotechnology (45.0% - color green). (For interpretation of the references to color in this figure legend, the reader is referred to the Web version of this article.)

It was found that 95% (n = 19) of the recovered assets are related to the chemical sector, while 5% (n = 1) correspond to the mechanical engineering sector. Regarding the technological field, 45% (n = 9) of the results obtained belong to the biotechnology field, 20% (n = 4) to the pharmaceuticals field, 20% (n = 4) to food chemistry, 5% (n = 1) to macromolecular chemistry and polymers, 5% (n = 1) to basic materials chemistry, and 5% (n = 1) to other special machinery. The distribution of the assets according to the technological field is illustrated in Fig. 3.

Based on these results, the protections of the patent modality at UnB are closely related to the technological field of biotechnology. According to recent literature, biotechnology is one of the leading technological trends related to innovation in the food area [7,23]. This data represents a strong alignment of UnB’s inventive activity with the technological trends in the food area and an excellent opportunity for the academic community involved in this technological field to direct its research lines to develop protectable assets. These protections can provide insertion in the food market and generate an expressive economic and social return for the University from partnerships aligned with consumer trends.

### 3.3 Workflow

Generally, the assets protected under a patent evolve during their validity period, passing through the main stages of filing, publication, technical examination, approval, or rejection. They may suffer requirements and be archived throughout this process and even after its conception [24]. Thus, as to their progress, we can classify patents and patent applications as provisionally archived (expectation of rights suspended for regularization of the application according to the requirement issued by the agency), definitively archived (patent application with its rights definitively suspended for failure to meet the requirement), in progress (patent application follows the usual process until the moment of technical examination); rejected (patent applications that passed the technical examination did not meet the patentability requirements, were denied and the protection process was terminated), extinct (granted patents that have already expired), granted (patent applications that were approved and the patent was granted) and withdrawn (patent applications that were removed at the applicant’s request) [8].

**Fig. 4:**
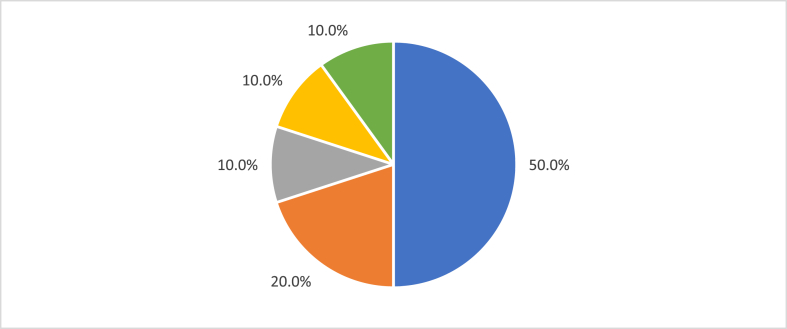
**–** Current status of the records. In progress (50.0% - color blue); Rejected (20.0% - color orange); Permanently archived (10.0% - color grey); Temporarily archived (10.0% - color yellow); Granted (10.0% - color green). (For interpretation of the references to color in this figure legend, the reader is referred to the Web version of this article.)

Considering these possibilities, the analysis of the status of the retrieved records revealed that 50% (n = 10) of the FUB food-related requests are in progress, i.e., they follow the ordinary course until the moment of technical examination, 20% (n = 4) of the requests have been rejected, 10% (n = 2) have been definitively archived, another 10% (n = 2) have an open archiving for lack of examination request, and another 10% (n = 2) have been granted. Fig. 4 shows the status of the records.

### 3.4 Deposits

**Fig. 5:**
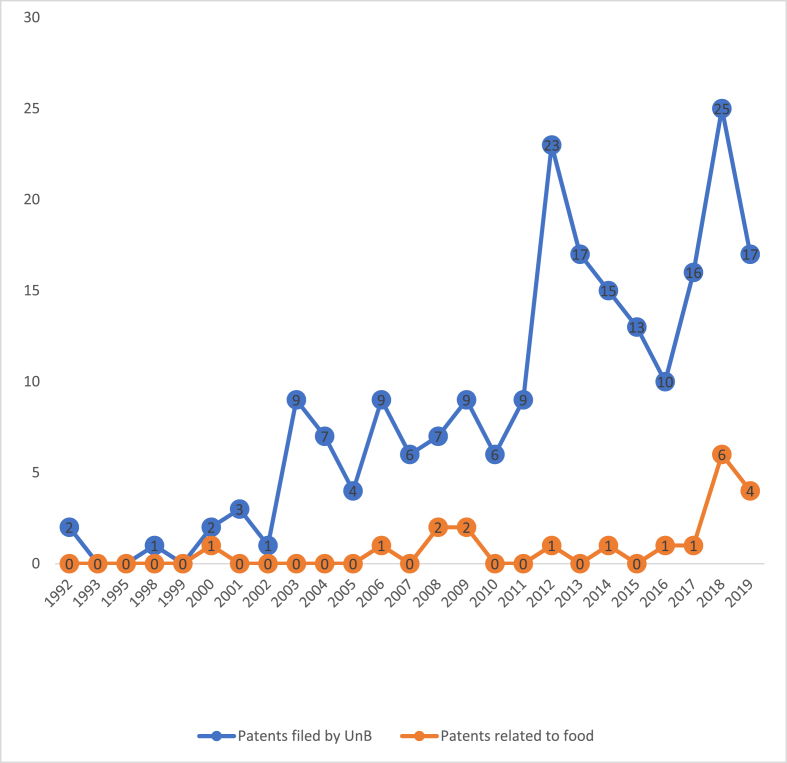
Evolution of the number of patent filings. Patents filed by UnB (color blue); Patents related to food (color orange). (For interpretation of the references to color in this figure legend, the reader is referred to the Web version of this article.)

2018 and 2019 represented the most significant number of food-related filings, accounting for 6 and 4, respectively. In the years 2000, 2006, 2008, 2009, 2012, 2014, 2016, 2017, 2018, and 2019, food-related patents accounted for 50%, 11%, 28.57%, 22.22%, 4.35%, 6.25%, 24%, and 23.53% of the patents filed by FUB, respectively. It is important to consider that the food-related applications from 2020 to 2021 were not included in the search because they are still in the confidentiality period. Fig. 5 presents the evolution of the number of food-related patent filings compared to the total number of filings made by FUB between 1992 and 2019.

The increase in the number of protections can be explained initially by the Program of Support for Restructuring and Expansion Plans of Federal Universities (Reuni), from 2007, which increased the number of teachers and students at UnB. Consequently, the institutional research and development efforts. The University’s outstanding effort in spreading the culture and knowledge about intellectual property has also contributed, resulting in more teachers, research projects, and scholars producing patent-protectable assets. Against this background, it is possible highlighting the meaningful exceeding of the goal of 15 patent deposits and registrations per year, established in the Institutional Development Plan 2018–2022 of the UnB, for the year 2020, within the objectives of the Department of Research and Innovation (DPI) to increase the number of UnB technology transfer [25].

The trend is that annually the number of protections grows, and consequently, the number of assets that need to be managed and monitored by the NIT/CDT increases. In this sense, the possible need for modernization of the management tools for these assets must be observed as the maintenance and training of a team of specialized collaborators. The NIT/CDT must be careful to maintain a balance between new demands and those already in progress at INPI so that there is no accumulation and stagnation of protection work, resulting in a freezing of the indicators over time [18].

### 3.5 Requirements

Fourteen requirements were issued. Most requirements were related to formal demands to determine whether access to the national genetic heritage. The requirements related to access to genetic heritage or associated traditional knowledge are particularly interesting for the food area. The formal requirement in question comes from Law No. 13,123/2015, which conditions the granting of patent applications obtained from access to genetic heritage or associated traditional knowledge to the registration or access authorization obtained from the Genetic Heritage Management Council (CGEN) [26].

Genetic heritage can be understood as the set of genetic information contained in plants, animals, and microorganisms, living or dead, and substances produced by them that occur natively or have acquired specific characteristics in the national territory. To access the genetic heritage, therefore, is to use the information contained in these organisms to study, test, or develop a marketable product or process, such as, for example, a food product [27].

The National System for the Management of Genetic Heritage and Associated Traditional Knowledge (SisGen) is the electronic tool used by CGEN to assist in the management of genetic heritage and associated traditional knowledge, where all research, experimental or theoretical, is required to be registered, carried out with Brazilian genetic heritage [28]. The knowledge generated from access to genetic heritage and associated traditional knowledge has substantial economic value. Therefore, registration ensures a return, conservation, and sustainable use of biodiversity and its commercial exploitation’s fair and equitable benefits [26].

### 3.6 Archiving

A total of 9 requests for archiving were issued, including six due to non-payment of annuity, due to payment of annuity after the deadline, or due to non-compliance with the requirement to complement the annuity payment, of which only one was not reversed and was permanently archived; and 3 for not requesting the examination request within the period provided for in the legislation, of which one was definitively filed and two, have until November 20, 2021, to request the withdrawal, upon payment of a specific fee for withdrawal and payment of the exam request. These filings will be considered definitive if unarchiving be not required and the examination request paid.

One must remember that managing protected assets requires weekly monitoring of the Industrial Property Journal (RPI) to follow all updates in the situations of patent applications, respond to demands, pay annuities, and request technical examination, among other administrative and technical tasks that require much time, dedication, and attention, and all these tasks must comply with the legal deadlines established under penalty of losing protection by filing. It is common for institutions to file a patent application and, in the future, not have the protections granted or to lose protections already granted due to a lack of follow-up and management of these assets. The more extensive the portfolio of intellectual property protections of an institution, the greater the management work, highlighting the importance of having a culture of innovation and efficient management of intellectual property and technology transfer [18].

To meet this challenge, it is of fundamental importance to have a team of qualified employees in the area. These professionals are not readily available on the market and must be trained in-house more often than not. For example, training a professional patent writer takes an average of two years. The experience of the NIT/CDT highlights the high turnover of these professionals, who, once trained, are absorbed by the market. Besides, the Career Plan for Technical-administrative positions in Education (PCCTAE) does not contemplate the profile required for performing these functions, which prevents them from being exercised by civil servants.

However, it is important to point out that not always the filings are due to a problem in managing these assets. Strategic and commercial issues discussed internally by the NIT/CDT may have influenced these filings so proceeding with a specific request was no longer interesting for the University. In this aspect, one must consider that the premise of requesting these protections is the generation of economic and social return to the UnB. It is necessary to analyze the context and investigate the motivations involved in the non-payment of the annuity or request for technical examination to know the reasons.

### 3.7 Rejections

The main reasons for rejections are the lack of compliance with the novelty or inventive step requirements. The non-compliance with the novelty requirement occurs when the claim is comprised state of the art, i.e., within everything that became accessible to the public before the filing date of the patent application, by written or oral description, by use or any other means, in Brazil or abroad, except for the conditions provided for in the legislation. The non-fulfillment of the inventive activity requirement occurs when a person skilled in the art derives the invention clearly or obviously from the state of the art [3].

Motivations were also identified related to the non-fulfillment of the invention unit concept; the industrial application requirement; the report not describing clearly and sufficiently the subject matter; the claims not being based on the descriptive report, characterizing the particularities of the application and defining, clearly and precisely, the subject matter of the protection; and the subject matter cannot be considered an invention or utility model since it is derived from plants and represent part of natural living beings found in nature.

### 3.8 Deferrals

**Fig. 6:**
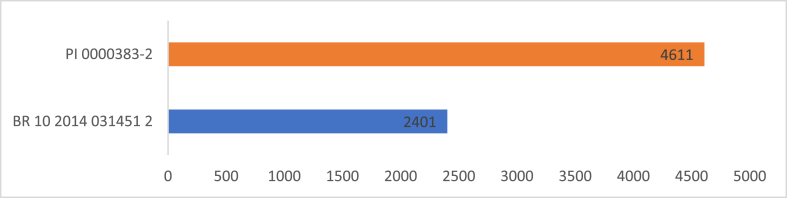
Time from filing to grant of the patents. BR 10 2014 031451 2 (2401 days – color blue); PI 0000383-2, (4611 days - color orange). (For interpretation of the references to color in this figure legend, the reader is referred to the Web version of this article.)

Fig. 6 illustrates the difference in time from filing to grant between granted patents. From the publication date to the grant of the two patents, the average time was 3506 days (±1563 days), or approximately nine and a half years. The validity period of the patents granted is 10 (ten) years counted as of 09/18/2012 for PI 0000383-2, i.e., valid until September 18, 2022, and 20 (twenty) years counted as of 12/16/2014 for BR 102014031451-2, i.e., valid until December 16, 2034 [29,30].

Thus, the entire process between the application and the conception can take, on average, ten years to happen because it follows a chronology in a waiting queue for the agency’s analysis, which finds itself with a lag in the number of examiners before the number of patent applications filed and, therefore, cannot keep up with the demand proportionally. This situation is known as Backlog and represents the liabilities generated by the delay in examining patent applications filed at the INPI [8].

To resolve this situation, INPI’s Patent Directorate (DIRPA) began the Backlog Combat Project aiming to substantially reduce the number of invention patent applications with required examination and pending decisions. The program’s goal was to reduce 80% of the 149,912 patent applications in two years that, on August 1, 2019, met conditions like: not submitted to the first technical examination carried out by the INPI; not the object of any priority examination request at the INPI; not containing a request for examination subsidies or subsidy opinion from the Brazilian Health Regulatory Agency (Anvisa); not having a corresponding request with anteriority searches carried out by Patent Offices of other countries, of international or regional organizations; and with filing date until 12/31/2016 [29,30].

The INPI’s strategy to achieve the proposed goals is to use the analysis results of patent applications in other countries and regions to support its technical analyses. This way, the INPI’s examiners can benefit from qualified technical work and save analysis time. According to data from the agency, until October 2021, 106,723 applications were decided or definitively shelved, representing a reduction of 71.18% of the Backlog [31].

### 3.9 Titularity

Regarding the depositors, 80% (n = 16) are deposits owned exclusively by the University of Brasilia Foundation (BR/DF), and 20% (n = 4) represent co-ownership with other organizations. The partner organizations included: *Coalhos Bio Paraná Ltda* (BR/PR), State University of Campinas - Unicamp (BR/SP), National Institute of Technology - INT (BR/RJ), and the Federal University of São João Del Rei (BR/MG).

Considering patent applications, patents, computer programs, and industrial design - together with cultivars' protections we obtain a very similar proportion since the protections made exclusively on FUB correspond to 73%. In comparison, 27% are co-owned by institutions, which means a considerable volume of protections that require formalizing the partnership in the food area [18].

In these cases, the ownership of the intellectual property and the participation in the results of the exploitation of the creations resulting from the partnership are foreseen in an intellectual property agreement. This type of agreement defines which of the institutions will manage that asset; the division of ownership among the joint owners; how the reimbursement of the fees paid for the maintenance of the technology will be done; the form of negotiations with possible interested companies; the responsibility for monitoring the improper and unauthorized use of the technology by unlicensed third parties, among other important aspects [18].

Identifying partner organizations in the protection of food-related technologies contribute to strategic decision-making in the sector and favors the establishment of a scientific and technological research network with independent inventors, universities, companies, and other entities interested in the development of technology, product, service, or process that can be protected by patent.

### 3.10 Partnerships between UnB and the food industry

The Brazilian food and beverage industry is the principal sector in the national economy and, worldwide, is the second-largest exporter of processed foods [4]. Although protection by intellectual property has a strong presence in the food industry, it may not be very effective [5]. Once formulations, methods, standards, techniques, and processes do not meet the requirements for patentability, industrial secrets are widely used for protection. A well-known example of trade secret use in the food area is the formula of the Coca-Cola® beverage [6]. An advantage of this protection is that there is no deadline to finish and that contracts guarantee the legal basement necessary for negotiations and technology transferences.

Despite these issues, although the scientific literature has long considered that the food industry have low innovation intensity, this scenario has changed in the last years [7]. In this context, patent protection for innovative products and processes generated within the food industry and related industries can represent a crucial market strategy [5]. In this context, it is essential to emphasize that partnerships with the food industry for research and development of new products and processes may raise assets eligible to be protected by intellectual property. The company may be interested in licensing protected technologies in partnership for use and commercial exploitation and even producing and marketing the technology itself. This fact demonstrates the importance of stimulating the approximation of the ICTs with industry and companies because these sectors will look to the ICTs for solutions to genuine demands of society and the chances of the research leaving the bench and becoming an innovation are certainly greater [18].

It is possible to transfer protected knowledge to third parties through partnerships, licensing, or even know-how transfer if patentability requirements cannot be met. One way to transfer technology is to purchase a license or patent from a university or research institute from a food company. At UnB, once Nupitec protects a technology, the Technology Commercialization Agency (ACT) starts its work to find possible partnerships for commercial exploitation [18]. Despite the low degree of maturity on food-related patents, the knowledge and know-how of UnB facilitate the transfer of technology.

Among the recovered technologies related to the food area that were successful in their commercial exploitation, we can highlight the PI 0601631-6 referring to “Jelly capsules of pequi pulp (*Caryocar brasiliense* camb) as a vitamin supplement, antioxidant and antimutagenic, a new nutraceutical. This technology was licensed from the work of ACT for the company RTK *Indústria de Cosméticos e Alimentos Naturais* LTDA (Naiak), generating recognition and financial return to the research developed within the UnB [32].

UnB also has co-ownership of some cultivars that are not protected with the INPI. In Brazil, the agency responsible for protecting cultivars, which is classified as *sui generis*, is Ministry of Agriculture, Livestock and Supply (MAPA) [33]. In some patent offices it is possible to protect cultivars by patents, but in Brazil this type of protection is not contemplated. Regarding the transfer of technology, the cultivars can be transferred through contracts that guarantee the part of benefit sharing and knowledge transfer [34].

The technology transfer indicators surveyed pointed out that the UnB has already carried out a total of 117 technology transfers, with 76 licensing patent applications or patents granted [15]. Patent applications and patents are the most licensed assets by UnB, equivalent to 65% of the technology transfers. Overall, the University has already received, like royalties, a total of R$34,870,715.71 since its first licensing [18].

## 4 Conclusion

Although patents are the primary modality of intellectual property protection at UnB, the participation of food-related assets is still not expressive. It was possible to identify that the trends and opportunities related to innovation in this area are mainly biotechnology. The industrial applications of these innovations are diverse, including their use in starch processing, in the manufacture of milk and dairy products, and as food additives, processing aids, food ingredients with functional and health claims, food supplements, foods with special porpoises, and other compositions claimed to be nutraceuticals.

Half of the UnB’s applications related to food are still in progress, and the other 20% (n = 4) were rejected, 10% (n = 2) were definitively filed, 10% (n = 2) are with filing requests open for lack of examination request and other 10% (n = 2) were granted. The years 2018 and 2019 represented the most significant number of food-related filings. The requirements issued for the processes were motivated by several issues, which stands out the need to inform whether there was access to genetic heritage. The motivations for achievements were related to the lack of payment of the annuity, non-compliance with requirements, and failure to request a technical examination. The lack of meeting the novelty or inventive step requirements while granted patents stood out as the reason for rejection. Currently, the two food-related patents granted are in force. The average time for the grant was nine and a half years. This delay results from the liabilities generated by the delay in examining the patent applications filed at the INPI, known as Backlog.

The results of the food-related patents were close to the overall development of the assets protected by the UnB, representing a considerable volume of protections that require the formalization of the partnership in the food area. It is possible to transfer protected knowledge to third parties through partnerships, licensing, or even know-how transfer if patentability requirements can not be met. The technology transfer indicators indicated that UnB has expertise in this area [18]. The case of technology transfer was highlighted to the RTK *Indústria de Cosméticos e Alimentos Naturais LTDA,* generating recognition and expressive financial return to the research developed at the UnB. The NIT/CDT should consider these data to improve its innovation culture and management of intellectual property and technology transfer. Future research will undoubtedly be necessary to follow the evolution of these processes at the INPI and investigate commercial opportunities for the UnB, giving continuity to the relevant theme that was intended to address.

## Funding statement

This research did not receive any specific grant from funding agencies in the public, commercial, or not-for-profit sectors.

## Author contribution statement

Rafael França Neves: Conceived and designed the experiments; Analyzed and interpreted the data; Wrote the paper.

Marileusa Dosolina Chiarello, Larisse Araújo Lima: Conceived and designed the experiments; Contributed reagents, materials, analysis tools or data.

Grace Ferreira Ghesti: Analyzed and interpreted the data; Wrote the paper.

## Data availability statement

Data included in article/supplementary material/referenced in article.

## Declaration of competing interest

The authors declare that they have no known competing financial interests or personal relationships that could have appeared to influence the work reported in this paper
